# Temporal variation in suicide in peri-urban Pretoria

**DOI:** 10.4102/safp.v63i1.5260

**Published:** 2021-05-11

**Authors:** Eric D. Onoya, Nokukhanya L. Makwakwa, David P. Motloba

**Affiliations:** 1 Department of Forensic Pathology, School of Medicine, Faculty of Health Sciences, Sefako Makgatho Health Sciences University, Pretoria, South Africa; 2 Department of Community Dentistry, School of Oral Health Sciences, Faculty of Health Sciences, Sefako Makgatho Health Sciences University, Pretoria, South Africa

**Keywords:** suicide, seasonality, diurnality; temporal variation, peri-urban, Pretoria

## Abstract

**Background:**

Suicide is a public health problem, and the third major cause of death in Indian, black and mixed race groups. In whites suicide is the second cause of death. The patterns of suicide vary by time of day, day of the week, month of the year and seasons. As a result of limited and inaccurate data, these variations have not been fully examined in the developing world. This study investigated the diurnality and seasonality of suicide in peri-urban Pretoria, as opposed to studies conducted previously in the country’s metropolitan.

**Methods:**

A retrospective analysis of suicides recorded between 2007 and 2019 was undertaken. Data were extracted from the forensic pathology department’s database (university mortuary).

**Results:**

Of the 1515 cases of suicides examined, majority were black Africans (95.9%), male (83.9%), aged 21–40 years (50.5%). Hanging was the most common method of suicide irrespective of demographics (72.8%). Diurnal suicide variations were distinct for men and women, occurring at (16:00–20:00) and (08:00–12:00), respectively. Suicide peaked on days preceding and after the weekend (Mondays and Fridays) and in warmer seasons (summer and spring)

**Conclusion:**

The overall patterns of suicide in peri-urban Pretoria, mimic local and global trends with regard to methods, demographics and temporal characteristics. The underlying mechanism for these trends is unclear requiring in-depth investigation in order to develop appropriate interventions.

## Introduction

Suicide, or intentional self-inflicted death is a complex phenomenon, which continues to be the subject of scientific research and philosophical debate to this day.^1,2^ Aetiological factors for suicide, are multifactorial and include social, psychological and physical factors, which impact heavily on the financial and psychosocial stability of the families and countries.^3,4^ Currently, global suicide is estimated to be around 800 000 cases, 10.7/10^5^ deaths per year.^5^ Suicide accounts for 1.4% of all deaths and is the 17th leading cause of death worldwide.^5^ It is predicted that by the turn of 2020, 153 million people will die of suicide, that is, one in every 20 deaths or one death every 40 s.^5^

Suicide rates vary widely amongst countries. In Europe, the former Soviet countries have the highest suicide mortalities, whilst China accounts for 30% of all reported suicide in the world.^6^ The continent of Africa has the lowest suicide rate despite higher overall mortality rate.^7,8,9^ In 2012, the mean suicide rate in South Africa was 17.2/10^5^ (8% of all deaths).^9^ Data on the incidence and patterns of suicide are limited across the continent, making the plans to mitigate the scourge of suicide challenging. Pretoria, metropolitan city recorded suicide rate of 25/10,^5^ compared with the national rate of 1.8/10^5^ from 1997 to 2000.^9^ Young adults and males are at an increased risk of committing suicide.^10,11,12,13^ Similarly, studies conducted in South India, United States and South Africa (SA) found suicide rates amongst adolescents to be higher in males than females.

In the past decade, the incidence of suicide at the Pretoria Medico-Legal Laboratory (PMLL) has remained unchanged. With regard to method used, there has been a substantial increase in hangings, whilst firearm-related suicides dropped significantly. Hanging remains the most common method of suicide globally, including South Africa and Pretoria.^11,12,13,14,15^ Firearm-related suicide was the second common cause of intentional death in Pretoria.^15^

Data from several observational studies indicate that suicide rates peak in spring.^6,16^ Recent data point to the flattening of spring peaks and emergence of smaller peaks in other seasons.^17,18^ Suicide rates varied by day of the week and time of day. More suicides occurred on Mondays or the first day before the weekend and during the early hours of the morning.^16,19,20,21,22^ This phenomenon is well documented in literature but not well described in Africa.

There is paucity of publications of the temporal variation of suicide in peri-urban areas of SA, this includes townships, rural areas and farms. The aim of the study was to describe the diurnality and seasonality of suicide in peri-urban Pretoria. We hypothesise, however that the pattern and distribution of suicide in peri-urban Pretoria will differ significantly from the national and global findings.

## Method and study design

A retrospective cross-sectional survey of records at the PMLL was conducted during the period 2007 to 2019. This facility undertakes medico-legal autopsies in the north and west regions of the Tshwane district. In South Africa, all reported cases on unnatural deaths to the Medico Legal Laboratory (forensic mortuaries) are contemporaneously handled by forensic medical practitioner and the investigating police officer. Autopsy findings, information from the scene of crime, medical and social history, and accounts from friends and family are used to determine the cause of death. Once the preliminary opinion has been given, the prerogative to classify the manner of deaths is the sole responsibility of the police and the prosecution. For the purposes of this study, all confirmed cases of suicide as captured in the National Injury Mortality Surveillance (NIMSS), were included in the study. All cases of unnatural deaths reported to the cases recorded before 2007 were not considered because of data capturing system faults at this Tshwane Forensic laboratory. Similarly, non-finalised and incomplete records were excluded. Information on demographics (age, race, gender), nature suicide, scene, date and time of day of suicide were extracted from the database.

All statistical analyses were undertaken using Statistical Package for Social Sciences (SPSS) version 24. The univariate analyses described the demographic profile of victims, method, time, day and season of suicide.The association between common methods of suicide and demographics, diurnality and seasonality were evaluated using Pearson’s Chi-square. Chi-square goodness-of-fit test was carried out to test the hypothesis that suicide in peri-urban Pretoria, differed from the metropolitan areas. For this purpose, our results on the common methods (hanging, poisoning and firearm use) in males and females were compared with the results from the Pretoria suicide study.^14^ The level of statistical significance was set at *p* < 0.05. Diurnality of suicide was reported based on six time intervals: night (24:00–04:00), early morning (04:00–08:00), late morning (08:00–12:00), early afternoon (12:00–16:00), late afternoon (16:00–20:00) and evening (20:00– 24:00).

### Ethical considerations

Approval to conduct this study was granted by the University’s Biomedical Research Ethics Committee (SMUREC/M/146/2020:PG) and Gauteng province.

## Results

### Demographic characteristics

A total of 1515 of 1610 medical records of suicide cases were included in this study for the period 2007–2019. The average age of the participants was 34.61 (±1399) years and a range of 10 to 91 years. The mean age for the males who committed suicide was 34.64 (13.76) years, which was not significantly different from that of the females, 34.34 (15.16) years. Independent of gender, suicide rate was highest amongst people aged between 21 and 40 years. There were more males (83.9%, *n* = 1271) than females (16.1%, *n* = 244), with the ratio of 5.2 male deaths to each female death. The ratio peaks to 7:1 in the 20s and plateaus until in the 5th decade. An overwhelming majority of victims were black African 1458 (96.2%), whites constituted 57 (3.8%) of the cases (Table 1).

**TABLE 1:** Demographic characteristics of suicides (2007–2016).

Age (years)	Number of cases (%)				Ratio	Total	
	Male		Female			*N*	%
	*n*	%	*n*	%			
≤ 20	142	11.2	43	17.6	3:1	185	12.2
21–30	356	28.0	51	20.9	7:1	407	26.9
31–40	305	24.0	52	21.3	6:1	357	23.6
41–50	174	13.7	28	11.5	6:1	202	13.3
51–60	121	9.5	28	11.5	4:1	1494	9.8
71+	173	13.6	42	17.2	4:1	215	14.2
**Total**	**1271**	**-**	**244**	**-**	**5:2**	**1515**	**-**

### Method of suicide by race, gender and age

The main method of suicide was by hanging, accounting for 1104 (72.9%) of all cases, followed by poisoning 197 (13.0%) and firearm or gunshot fatalities 144 (9.5%). In a minority of cases, vehicle impact claimed 22 (1.5%) lives and burns and electrocution, 28 (1.8%). Curious findings are observable when examining suicide by race, gender and age. Significantly, more white people ingested poison or used firearms to commit suicide, whilst black people hung themselves in a majority of cases (*p* = 0.000). Proportionally, three times more females committed suicide by poisoning than males. The age predilection of a method for suicide were opaque and insignificant (*p* = 0.136). However, the elderly (over 70 years), committed suicide through poisoning than other methods (Table 2). A comparison between our study’s findings and the Pretoria investigation^14^ indicates that more males committed suicide through hanging (79.5% vs. 47.9%). However, for firearms and poisoning related suicide, the proportion of males was comparatively reduced, (10.4% vs. 23.8%) and (10.1% vs. 12.6%), respectively. The association of suicide method and gender differed significantly from Pretoria^14^ study (*X*^2^ = 266.8, *df* = 2; 0.000). Similar significant differences were recorded amongst males and the female groups (X^2^ = 48.75, *df* = 2; 0.000). The rates of suicide by poisoning were similar (32.9% vs. 31.9%), lower for firearm use (7.2% vs. 15.0%) and higher for hangings (59.9% vs. 28.8%).

**TABLE 2:** Common method of suicide by race, gender and age.

Variable	Category	Hanging		Poisoning		Gunshot		*X* ^2^	*p*
		*n*	%	*n*	%	*N*	%		
Race	White people	22	43.1	12	23.5	17	33.3	40.7	0.000
	Black people	1078	77.8	182	13.1	126	9.1		
Gender	Male	970	79.5	123	10.1	127	10.4	82.2	0.000
	Female	133	59.9	73	32.9	16	7.2		
Age	≤ 20	145	81.0	21	11.7	13	7.3	14.9	0.136
	21–30	306	79.8	47	12.1	35	9.0		
	31–40	251	74.1	52	15.3	36	10.6		
	41–50	140	72.1	30	15.5	24	12.4		
	51–60	111	80.4	10	7.2	17	12.3		
	71+	150	73.5	36	17.6	18	8.8		

### Variation in suicide by time of day

Diurnal variations in the distribution of major methods of suicides is apparent in both genders and age groups. The fewest suicides occurred between (00:00–04:00) in males and (20:00–24:00) in females. A significant increase in suicides amongst males starts early in the morning (04:00–08:00), reaching the peak in the late morning (08:00–12:00), followed by a steady decline and another increase in the late afternoon (16:00–20:00). This trend is more pronounced between the ages of 21 and 40 years, especially in males. The suicide rate in females, remained relatively steady until a sharp increase or peak between 16:00 and 20:00. Strikingly, proportionally more females than males aged 21–30 years commit suicide between 00:00 and 04:00 (Table 3).

**TABLE 3:** Distribution of most common suicide by time of day across gender and age.

Gender	Total	00:00–04:00	04:00–08:00	08:00–12:00	12:00–16:00	16:00–20:00	20:00–24:00	*p*
**Male**	-	112	206	270	233	247	153	-
	≤ 20	10	18	40	19	34	16	0.000
	21–30	32	62	74	60	61	53	0.004
	31–40	30	49	52	64	65	31	0.000
	41–50	14	34	38	33	33	18	0.005
	51–70	7	17	32	25	26	12	0.000
	71+	19	26	34	32	28	23	0.328
**Female**	-	38	34	34	41	50	31	0.291
	≤ 20	7	4	4	10	11	5	0.232
	21–30	10	6	7	9	10	6	0.814
	31–40	8	9	6	9	12	6	0.694
	41–50	2	3	4	6	5	5	0.761
	51–70	5	3	5	5	7	2	0.632
	71+	6	9	8	2	5	7	0.416

### Suicide deaths by day of the week

**FIGURE 1:**
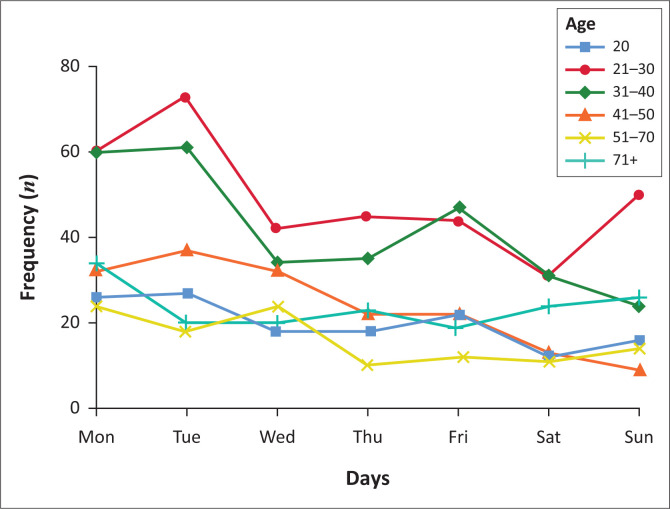
Daily variations in suicide by age in males.

**FIGURE 2:**
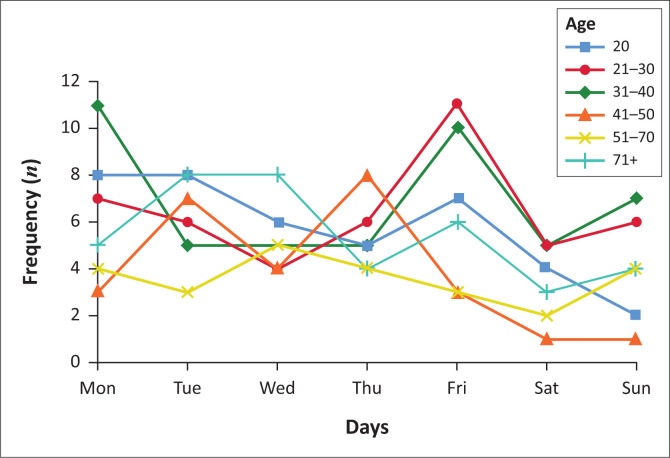
Daily variation in suicide by age in females.

The distribution of suicide deaths by the day of the week varied by gender and age. The patterns were consistent, despite the observed significant differences in males compared with females. Suicide was commonly committed on Mondays and Fridays by all genders, especially amongst the younger age-groups (≤ 40). Additional suicide peaks were observed on Tuesdays and Sunday for males (Figure 1) and on Wednesdays and Thursdays for females (Figure 2).

### Suicide by seasons of the year

**FIGURE 3:**
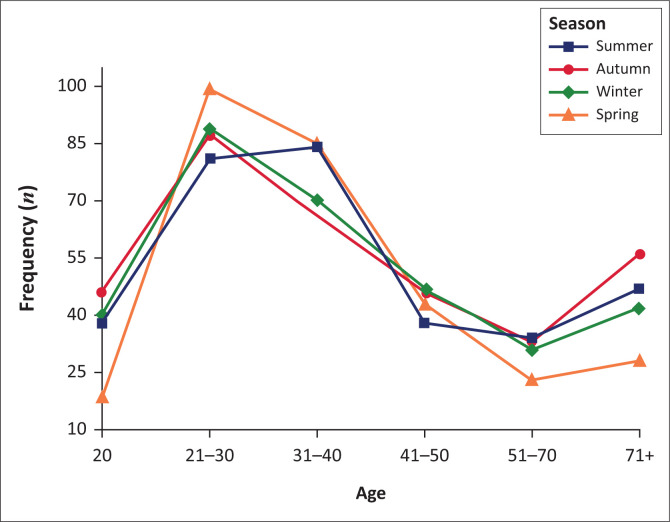
Seasonal variation in suicide by age in males.

**FIGURE 4:**
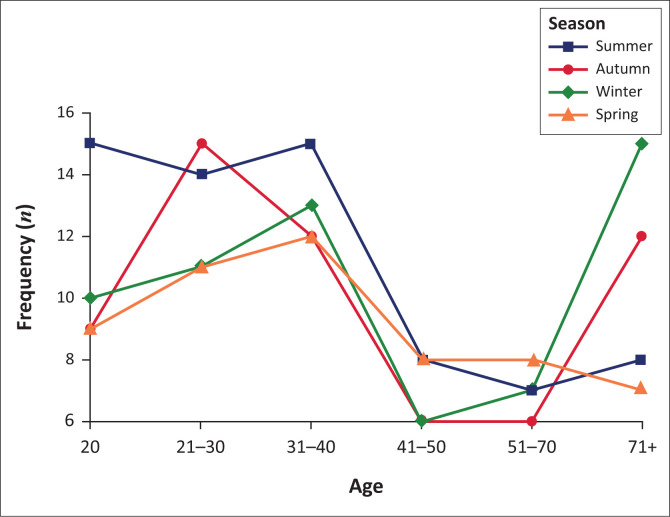
Seasonality of suicide by age in females.

Overall, there was no observable difference in the distribution of suicide by season. The suicides peaks were almost similar, except for a slightly pronounced increase in spring. (*X*^2^ = 2.51, *df* = 3; 0.47). Gender differences were insignificant at *p* = 0.502 and 0.73, respectively, despite suicide peaks in autumn and summer for males (Figure 3); summer and winter in females (Figure 4). There was a marked increase in incidents of suicide in summer amongst males aged between 21 and 40 years. Females younger than 40 years committed suicide in spring and those beyond 70 years in winter.

## Discussion

The results show males are more likely than females to commit suicide using violent means, that is, hanging and firearms compared with females who die of poisoning. The findings confirm that suicide occurs more frequently during the day; on days preceding or following the weekend, that is, Mondays and Fridays and in the summer and spring seasons. Demographic differences in suicide rates were related to access, socialisation, culture and other socio-behavioural factors.

### Demographic variations in suicide

The majority of suicides occurred amongst black Africans than whites. The over-representation of blacks (95.64%) in this study confirms the distinct population dynamics in peri-urban areas of Pretoria compared with the metropolitan areas.^11,14,23^ In terms of age-related variations, the results show that a majority of suicides occur amongst the 21–40 year olds, which is comparable to South African studies.^11,12,14^ Suicide in this age group is attributed to ever worsening socio-cultural and economic challenges in this areas. High levels of poverty, unemployment, indebtedness in townships may contribute to psychological problems including suicide ideation and death. We found a comparatively larger proportion of elderly victims of suicide 14.19% compared with 6.4% in a Pretoria^14^ and 2.6% in a Bloemfontein study.^23^ We theorise, that black Africans in the townships are unable to preserve strongly and firmly integrated extended family structures.

As a consequence, the elderly, might not be surrounded by family and friends to provide support. At this age, the elderly would have suffered multiple loss, which adds to further isolation and social deprivation. The experience of loneliness remains the primary risk factors for suicide amongst the elderly.^24^ Declining physical, mental and emotional health also contribute to the increased occurrence of suicide in the elderly.

Our findings concur with available literature that overwhelmingly more males commit suicide than females.^11,12,25^ Socialised masculinity encourages men to be ‘strong’ and not express their emotions because it is ‘weak’ and socially unacceptable.^25^ Men experience problems as women do, but are more likely to procrastinate and not to seek help. Ultimately, most males resort to substance abuse, especially alcohol, as a means to deal with stressful situations. However, alcohol exacerbates depression and increases impulsive behaviours, including committing suicide.^26^ Alcoholism is a known risk factor for suicide.^27^

### Variations in methods of suicide

Suicide research has not fully explained why victims choose a particular method of death. However, it is reported in published literature that males are most likely to use violent methods to commit suicide, whilst females resort to poisoning and other less violent means. Studies undertaken in South African metropolitan cities^11,12^ and Pretoria^14^ confirm this finding. The choice of methods is attributable to the availability of the means, familiarity and the ease of execution.^13,28,29^ Males have access to, and are able to use firearms, whilst women resort to poisoning and other available methods. This could also explain the race and age differences in the rates of suicide in our study. Whites who are largely based in the farms and the young would have better opportunities to access and use firearms. Similarly, more white women than blacks will use firearms given greater access to these means of committing suicide.

Another explanatory theory is the Beautiful Corpse thesis, which asserts that women are more likely than men to commit suicide in ways that minimise disfigurement of the face and head.^13,28^ Hence, methods such as a poisoning, use of carbon monoxide gas, instead of using firearms or jumping from heights would be preferable for this cohort. Socialisation of physical beauty and its reinforcement by social media, strengthen the notion that women should be attractive and beautiful even in death. Payne et al., provide an alternative theory for why women do not choose violent methods of suicide.^30^ Their argument is that women are ‘Relational Beings’ and are out of consideration for family, friends and other relations, less likely to disfigure their corpses. The Relational Being theory, suggests that compassion rather than familiarity or access to means is the primary driver of the choice of method of suicide.^30^ Accordingly, women are geared to preserve their physical image, thereby protecting their loved ones from trauma arising from physically disfigured body. An emerging alternate explanation is that women use less violent methods because they are less intentional about dying than men.^31^ Whilst this study did not assess this variable, we found that twice as many females than males (12.8% and 5.7%), died at the hospital or emergency room. This could be indicative of the gender paradox, according to which, 2–3 times more females attempt suicide and 2–3 times more males die from suicide.^31^ Further research on the intent and completion of suicide is critical to distinguish between those who want to die but fail and those who do not intend to die but succeed. This will have implications on the veracity of this theory and how it applies in peri-urban Pretoria.

### Temporal variations of suicide

This study reported variations in suicide by time of day, day of the week and season of the year. It is generally reported that suicides are attempted or committed in days preceding the weekend or after the weekend. It is theorised that the depressed individuals, often put their hopes in the weekends to usher in a ‘fresh start’, which unfortunately never materialise. Feeling distraught the affected individuals may experience major disappointment and depression, as life circumstances do not change significantly to provide hope in the future. These life changes vary by moment of the day (diurnal variations), change with every day of the week and as seasons pass. Some moments of the day, some days and seasons are dark and depressing, blue or bright, which could lift spirits or exacerbate gloominess. Suicidal ideation and finally suicide maybe associated with the changing times.

#### Diurnal variations of suicide

Suicide occurred in the late morning and afternoon (08:00–12:00) and (16:00–20:00), for males and females, respectively. Suicide is most prevalent when people are actively alone. We hypothesise that these times present opportunities for victims to be alone and isolated with minimal interference or support. In the context of peri-urban living, late mornings present opportunity to commit suicide for the majority of people who stay at home or the unemployed. The late afternoons represent the time when largely employed males commit suicide, preferably outside their place of residence. Males are likely to report from work and leave their homes to commit suicide elsewhere. Our findings are similar to reports by several authors.^16,20,21,22^ Contrary findings indicate that suicide rates are greatest at night, ‘when reason sleeps’, the so-called nocturnal wakefulness.^32,33,34^ Insomnia and other sleep disturbances increase the vulnerability to suicide ideation, attempt and eventual deaths.^2^ Being awake in the early hours of the morning when it is not biologically ideal or desired, results in diminished cognitive and other brain functions or ‘hypofrontality’, which increases risk for suicide ideation and death.^34^

#### Daily variation of suicide

The increased suicide rates on Monday and Friday corroborates the blue Monday myth.^17,35^ On these days, people feel depressed because the festivities are over, finances depleted, friends are gone and they are all alone. During these periods, no support is at hand, and the activities that mask the turmoil are over and victims have to deal with their depression and all head-on. Most persons might not have the capacity and willingness, but the opportunity to end their lives. This argument whilst plausible is a huge causal leap between feeling blue and depressed to committing suicide. In some instances, it has been termed pseudo-science that trivialises depression and undermines efforts to understand the diurnality of suicide. There is consensus in literature about Mondays as the peak day for suicides. Friday and other days are reported discriminately without any discernible pattern.

#### Seasonal variations of suicide

There was no seasonal variation in suicide in this study. This finding supports numerous accounts of the reducing fluctuation or disappearance of seasonality of suicide as evidenced by lower peaks, in summer and spring.^18,36^ Currently no plausible hypothesis has been posited to explain this emerging phenomenon. It is instead argued that connectedness reduces suicide and seasonal variation.^37^ Accordingly, the widespread penetration and adoption of technology facilitates connection between people anywhere and anytime. This results in reduced communication gap, which lessens feelings of isolation, loneliness and depression, which explains to some degree, the diminishing seasonal variation.

### Strengths and limitations of the study

This study has a few strengths, firstly, this research consists of the analysis of the largest data set in this setting. Secondly, this research provides estimates on the temporal variations of suicides, this information is critical in understanding the burden of suicide in Pretoria. Thirdly, this study adds to few studies that sought to investigate the patterns of suicide outside the metropolitan cities of South Africa. Fourthly, the study has reasonably addressed possible limitations that could invalidate our findings.

The results of this study are consistent with available literature, yet caution needs to be exercised in the interpretation and use of these findings. Although the sample size is relatively adequate (reasonable studies, *n* ≥ 1000), methodological limitations that are inherent in suicide studies are applicable in this research as well: (1) the use of time of death as a proxy for the time of suicide for some forms of suicide may result in inaccuracies. For example, in cases of non-violent suicide such as poisoning, it is difficult to establish the time of death with precision. The time between the act and eventually death may vary from minutes to hours. Majority of suicides in our study are by hanging (72.9%) and gunshot (9.5%), which are amongst the quickest forms of death. It is likely that the majority of deaths occurred shortly after the act, which reduces the possible temporal bias. (2) Another inherent bias is ascertaining whether the death is a suicide (intentional) or unintentional, which can affect the extrapolations of our findings. This threat to validity is not confined to our study alone, but pervades suicide studies. The use of NIMSS and robust processes to determine the cause of death in South Africa has significantly reduced misclassification. Underestimation of suicidal deaths is a worldwide phenomenon, resulting from strong social, religious and cultural beliefs, which discourage truthful disclosure. Commonly misrepresented suicides are poisoning, overdose and vehicle-related accidents,^38^ which constitute an insignificant proportion of deaths in this study. (3) The number of unrecorded suicide in the peri-urban Pretoria is unknown and could be higher than the registered figures, thus changing the observed patterns. Opportunely, NIMMS data, especially in Tshwane, has demonstrated to have high levels of sensitivity, specificity and predictive value across different demographic groups.^39^ Therefore, the under-reporting of suicide cases might not have a significant effect on the diurnal and seasonal variations in our study.

### Implications

This study is descriptive is nature, hence causality cannot be inferred. However, these findings are important in highlighting peculiar factors that pervade suicide behaviour in townships and peri-urban settlements in Pretoria. The existence of a temporal variation in suicide is indicative of better surveillance of individuals at risk of suicide and greater attention to chronobiological factors leading to suicide ideation and death.

Understanding the patterns and risk factors for suicide in these areas can assist to develop interventions that are specific and appropriate to these settings and the prevailing sociodemographic and economic factors. This study should influence policies aimed at reducing the rates of suicide in Pretoria and similar areas in the country.

## Conclusion

The hypothesis that the patterns of suicide in peri-urban Pretoria were significantly different from the rest of the country was disproved. We conclude that overall, suicide trends are similar across the country including peri-urban Pretoria. Males have a high suicide preponderance, whilst females are more likely to die by poisoning. Suicide is committed most frequently in summer and spring, on Monday and Friday and during the daytime. Socioeconomic and behavioural risk factors for suicide in these settings are not well understood. Comprehensive analytical studies are needed to investigate these risk factors.
